# *Clostridioides difficile* outbreak detection: Evaluation by ribotyping and whole-genome sequencing of a surveillance algorithm based on ward-specific cutoffs

**DOI:** 10.1017/ice.2023.113

**Published:** 2023-12

**Authors:** Jon E. Edman-Wallér, Michael Toepfer, Johan Karp, Kristina Rizzardi, Gunnar Jacobsson, Maria Werner

**Affiliations:** 1 Centre for Antibiotic Resistance Research (CARe), Department of Infectious Diseases, Institute of Biomedicine, Sahlgrenska Academy, University of Gothenburg, Gothenburg, Sweden; 2 Department of Clinical Microbiology, Sahlgrenska University Hospital, Gothenburg, Sweden; 3 Clinical Microbiology, Unilabs AB, Skövde, Sweden; 4 Department of Infectious Diseases, Skaraborg Hospital, Skövde, Sweden; 5 Department of Microbiology, Public Health Agency of Sweden, Solna, Sweden; 6 Department of Infection Prevention and Control, Södra Älvsborg Hospital, Borås, Sweden

## Abstract

**Objective:**

We evaluated the performance of an early-warning algorithm, based on ward-specific incidence cutoffs for detecting *Clostridioides difficile* transmission in hospitals. We also sought to determine the frequency of intrahospital *Clostridioides difficile* transmission in our setting.

**Design:**

Diagnostic performance of the algorithm was tested with confirmed transmission events as the comparison criterion. Transmission events were identified by a combination of high-molecular-weight typing, ward history, ribotyping, and whole-genome sequencing (WGS).

**Setting:**

The study was conducted in 2 major and 2 minor secondary-care hospitals with adjacent catchment areas in western Sweden, comprising a total population of ∼480,000 and ∼1,000 hospital beds.

**Patients:**

All patients with a positive PCR test for *Clostridioides difficile* toxin B during 2020 and 2021.

**Methods:**

We conducted culturing and high-molecular-weight typing of all positive clinical samples. Ward history was determined for each patient to find possible epidemiological links between patients with the same type. Transmission events were determined by PCR ribotyping followed by WGS.

**Results:**

We identified 4 clusters comprising a total of 10 patients (1.5%) among 673 positive samples that were able to be cultured and then typed by high-molecular-weight typing. The early-warning algorithm performed no better than chance; patient diagnoses were made at wards other than those where the transmission events likely occurred.

**Conclusions:**

In surveillance of potential transmission, it is insufficient to consider only the ward where diagnosis is made, especially in settings with high strain diversity. Transmission within wards occurs sporadically in our setting.

*Clostridioides difficile* infections (CDIs) are common in hospitals, with a mix of sporadic cases of unknown source and cases that are transmitted mostly indirectly from patient to patient.^1,2^ Sweden has a high incidence of CDI compared to the rest of Europe, despite a prudent use of antibiotics and sporadic occurrence of the epidemic *C. difficile* strain ribotype (RT) 027.^3,4^ National surveillance data have shown a high diversity of ribotypes, and only a few outbreaks have been described in recent years.^4,5^ However, the incidence of transmission of *C. difficile* in Swedish hospitals in the absence of apparent outbreaks has hardly been studied.^6,7^

Indirect transmission between patients can lead to outbreaks with severe consequences.^5,8^ Although whole-genome sequencing (WGS) together with epidemiological data is the best way of identifying transmission, it is costly and unpractical to perform on all positive *C. difficile* specimens in clinical practice. Simple tools are needed to screen for CDI cases to suspect as part of transmission chains and further examined by molecular typing and/or WGS.

We have previously proposed a surveillance strategy in which the cutoffs for suspecting *C. difficile* outbreaks are based on historical ward incidence and the Poisson distribution.^9^ We performed this study with the primary aim to evaluate this strategy. Our secondary aim was to describe the extent to which transmission of symptomatic CDI occurs between patients within wards in a Swedish setting.

## Methods

### Setting

The study was conducted in 2 major and 2 minor secondary-care hospitals with adjacent catchment areas in western Sweden, with a total population of ∼480,000 and encompassing 1,000 hospital beds. The hospitals are served by 2 microbiology laboratories that also serve primary care within the catchment area; thus, both primary-care tests and hospital tests were included in the study. No apparent outbreaks of CDI have occurred at the hospitals in recent years. The number of hospital beds per person (2.1 per 1,000 inhabitants) is average for Sweden but low compared to the rest of Europe.^10^ Antibiotic consumption is low, especially in primary care,^3^ and rates of antimicrobial resistance are comparably low.^11^ Infection control routines for CDI patients include care for symptomatic patients with contact precautions in single rooms with private bathrooms until 48 hours after diarrhea has resolved, daily disinfection of patient-adjacent areas with an oxidative agent (Incidin, accelerated hydrogen peroxide; Ecolab, Saint Paul MN), and handwash followed by alcohol handrub after patient care. The coronavirus disease 2019 (COVID-19) pandemic occurred during the study period, leading to shifts in the patient populations of several wards (eg, twice as many intensive care patients as usual during peaks and some wards being repurposed as pandemic wards). The pandemic also entailed a general focus on preventing COVID-19 transmission, including an increased use of personal protection equipment.

### Transmission

To detect transmission events between symptomatic patients, all positive CDI samples were collected for a 2-year period (January 1, 2020–December 31, 2021). Each case was assigned a study identifier with 1 letter and 3 digits; the letter was the first letter in the city where the laboratory was located, and the digits were consecutive numbers starting at 001. At both laboratories, a standalone PCR test for *C. difficile* toxin B was used for diagnosis: BDMax (Becton, Dickinson and Company, Franklin Lakes, NJ) was used at 1 hospital and Amplidiag *C. difficile* +027 (Mobidiag, Espoo, Finland) was used at the other. Tests were ordered by the attending physician based on clinical suspicion and was only performed on loose-stool samples. No screening practices were in place. Positive tests were included in the study and were cultured and typed using MALDI-TOF high-molecular-weight (HMW) typing,^12^ a typing method that correlates to PCR ribotyping but with lower resolution. Medical records of all patients were examined to determine at which wards every patient had been cared for during the 60 days before and 30 days after the test was taken. Patients with the same HMW type and a history of care at the same ward within 30 days of each other were further investigated for possible transmission. In these cases, PCR ribotyping and WGS were performed. Cases in which the number of single-nucleotide variants (SNVs) differed by 2 or less were considered confirmed transmission events, based on previous studies on the *C. difficile* mutation rate and expected variations in connected cases.^1,13^

### Poisson early-warning algorithm

This algorithm was based on the definition of an outbreak as “more cases of a disease than expected in a specific location over a specific time period,”^14^ where the location was a given hospital ward, and the period is 30 days. For each ward, historical incidence (cases per month among samples sent from that ward) of CDI from July 2018 to December 2019 was used together with the Poisson distribution to determine a cutoff for early warning, as previously described.^9^ For a ward with a mean number of cases of, for example, 1 per month, the probability of 4 or more cases to occur by chance during a 30-day period would be 1.9%. The algorithm triggers an early-warning alert when the observed number of cases is expected to occur in <5% of 30-day periods. The maximum number of cases in a 30-day period accepted without an early warning varied between 1 and 3 at different wards.

### Two-case algorithm

For comparison, we also evaluated a simpler 2-case algorithm, based on 2 cases occurring at the same ward within 30 days of each other, regardless of the historical ward incidence. This algorithm was based on the definition of an outbreak as “2 or more cases of the same infection related in time and place,” which is often used in practical infection prevention and control guidelines.^15^

### Patient data

In addition to ward history, information on age, sex, and 30-day all-cause mortality was collected.

### PCR ribotyping and Whole Genome Sequencing

PCR ribotyping of *C. difficile* isolates was performed using capillary PCR ribotyping as previously described.^12^ All isolates belonging to the same ribotype were further analyzed by WGS to investigate potential transmission events. WGS was performed on the Ion Torrent platform at the Public Health Agency of Sweden, followed by SNV analysis as previously described.^16^ A mean coverage of 41.1x (standard deviation, 17.0x) was obtained.

### Statistical methods

The ability of the early-warning algorithms to detect transmission events was evaluated by determining the sensitivity, specificity, positive and negative predictive values with 95% confidence intervals (CI) based on the binomial distribution. This calculation was based on each case and whether it was part of a transmission event or not. Data are presented as mean (SD) for continuous variables that are approximately normally distributed and number (%) for categorical variables. Comparisons between groups were performed using the Student *t* test for continuous variables and the Fisher exact test for categorical variables. The Simpson index (D) was calculated using the formula 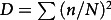 and the Simpson diversity index was calculated using 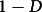 . Microsoft Excel 365 software (Microsoft, Redmond, WA) and SPSS version 28 software (IBM, Armonk, NY) were used for statistical calculations and graphs.

### Ethical considerations

The study was reviewed by the regional ethics board of Göteborg (no. 2019-03298).

## Results

Overall, 4 clusters comprising a total of 10 patients (1.5%) were confirmed with WGS among 673 positive samples that were culturable and typable by HMW typing (Table 1). A further 141 positive samples (14.3%) for which culture and/or typing failed were excluded from all analyses. The mean age of patients involved in clusters was 77.2 years (SD, 22.1); 7 patients (70%) were women, and 1 patient (10%) died within 30 days of the test. Among patients that did not belong to a cluster, the mean age was 67.5 years (SD, 20.3); 357 patients (54.3%) were women, and 60 patients (9.2%) died within 30 days. None of these differences were statistically significant.

**Table 1.** Ward History and Early-Warning Algorithm Alerts for Patients in the Confirmed Clusters

Cluster (no. of cases, ribotype, ST)	Patient	SNV^2^ Differences	Shared Ward	Days Between Shared Ward	Ward Where Test Was Taken	Poisson Alert	2-Case Alert
A (3 cases, RT014, ST 2)	S088	0–1	Surgical (general)	57	Surgical (gastrointestinal)	…	…
	S117				Medical (general)	…	…
	S245			223	Infectious diseases (outpatient)	…	…
B (2 cases, RT020, ST 2)	S225	0	Medical (nephro)	0	Medical (nephro)	Medical (nephro)	Medical (nephro)
	S238				Medical (general)	…	Medical (general)
C (3 cases, RT001, ST 3)	B125	0	Medical (nephro)	12	Infectious diseases	Infectious diseases	Infectious diseases
	B145		Medical (nephro)		Medical (nephro)	Medical (nephro)	Medical (nephro)
	B166		…	…	Neurology (stroke)	…	…
D (2 cases, RTx231, ST 11)^a^	B209	1	Surgical ward (mixed)	0	Medical (lung)	…	Medical (lung)
	B225				Medical (general)	…	…

Note. ST, sequence type; SNV, single-nucleotide variation.

a The “x” indicates that international nomenclature for the ribotype is missing.

Cluster A consisted of 3 patients with a shared history at a general surgery ward, a high-turnaround ward to which surgical patients are typically admitted from the emergency department. Patient S088 was diagnosed with CDI shortly after staying at the shared ward, and patient S117 was exposed to the shared ward 57 days later and was subsequently diagnosed after transfer to a medical ward. Patient S245 was exposed to the shared ward >7 months later and was subsequently diagnosed at the infectious disease outpatient clinic.

Cluster B consisted of 2 patients who were cared for at a nephrology ward at the same time. Although patient S225 was diagnosed at this ward while admitted, patient S238 was diagnosed 35 days later at another medical ward after being discharged and readmitted.

Cluster C consisted of 3 patients, 2 of whom were cared for in close temporal proximity to one another in a ward for patients with renal and gastrointestinal diseases. Patient B125 was diagnosed with CDI at the infectious diseases ward prior to being transferred to the shared ward, to which patient B145 was transferred 12 days after patient B125 had been discharged. The third patient, B166, was diagnosed 20 days after patient B145 but had no shared ward history with the other 2 patients.

Cluster D consisted of 2 patients who received care at the same surgical ward at the same time. This ward admits a mix of surgical patients with upper gastrointestinal disease; ear, nose, and throat disease; vascular disease; and urologic disease. Patient B209 was diagnosed with CDI 12 days later after being transferred to a pulmonary ward. Patient B225 was diagnosed 36 days later at a general medical ward after being discharged and readmitted.

**Figure 1.**
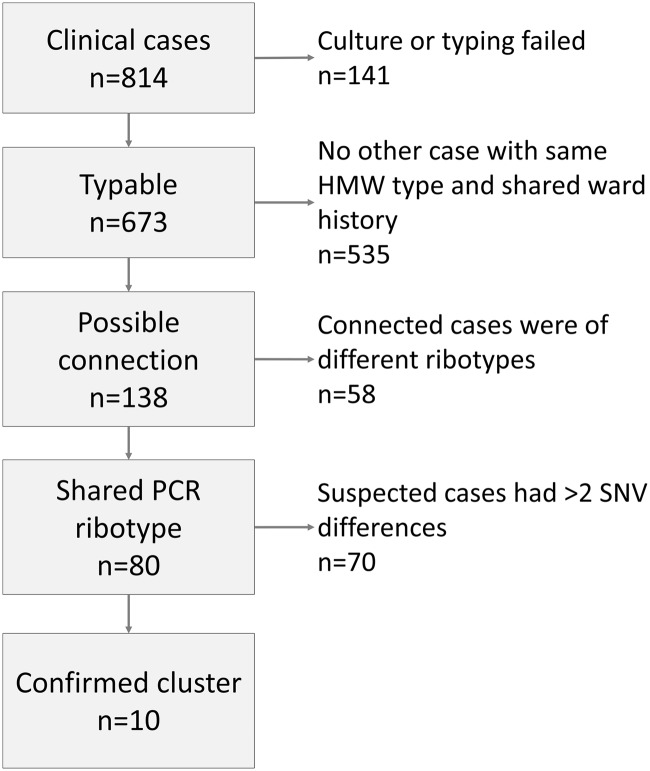
Flow chart depicting the steps for identifying transmission clusters.

Isolates were distributed over 34 different HMW types, with a Simpson diversity index (1 − D) of 0.9. HMW14 was the most common type and correspondes, among others, to RT014 and RT020, which are 2 of the most common ribotypes in Sweden.^4^ Two isolates (0.3%) were HMW24, corresponding to RT027. PCR ribotyping was performed on 138 isolates, in which 2 or more patients had a shared ward history and the same HMW type. These isolates belonged to 33 different ribotypes of which RT014 was the most common, followed by RT020. WGS was performed on 80 isolates (Fig. 1).

**Figure 2.**
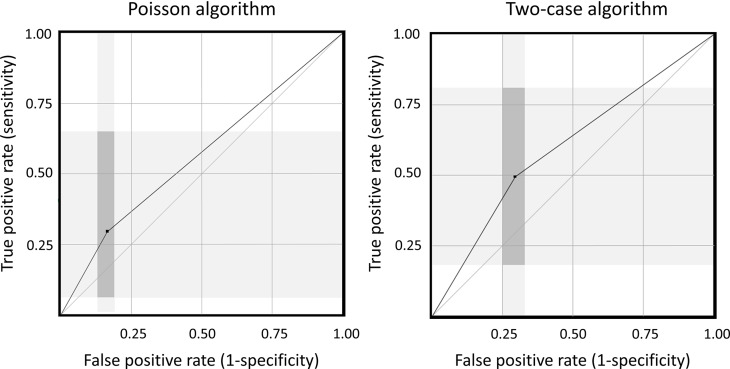
Performance of the 2 evaluated algorithms for detecting transmission events. Note. Grey areas are confidence intervals; black dots are point estimates.

The Poisson early-warning algorithm identified 39 possible clusters with a total of 111 patients, and 3 patients were part of confirmed transmission events: sensitivity, 30.0% (95% CI, 6.7%–65.3%); specificity, 83.7% (95% CI, 80.7%–86.4%); positive predictive value, 2.7% (95% CI, 0.6%–7.7%); and negative predictive value, 98.7% ((95% CI, 97.5–99.5%). The 2-case algorithm identified 73 possible clusters, with a total of 202 patients, of whom 5 patients were part of confirmed transmission events: sensitivity, 50.0% (95% CI, 18.7%–81.3%); specificity,70.3% (95% CI, 66.7%–73.7%); positive predictive value, 2.5% (95% CI, 0.8%–5.7%); and negative predictive value, 98.9% (95% CI, 97.5%–99.7%). Figure 2 shows a visualization of the sensitivity and specificity data.

Within the confirmed clusters, all cases were diagnosed at different wards. The ward where the test was taken was, in most cases, different than the ward where transmission likely occurred. The time from care at the shared ward to diagnosis ranged between −3 days and 36 days with a median of 5 days. A negative number means that the patient had symptomatic disease before being admitted to the shared ward where transmission likely took place, which implies that the patient was the source of transmission.

## Discussion

Symptomatic transmission from patient to patient within wards occurs regularly in our setting, albeit in a limited number of cases. Of 80 patients suspected to be part of clusters based on PCR ribotyping and ward history, only 10 could be confirmed by WGS. This indicates that PCR ribotyping does not have sufficient resolution to confirm transmission, even in high-diversity settings. Importantly, the ward in which transmission likely occurred was different from the ward where the CDI test was taken, in almost all cases. This finding suggests that surveillance based on ward incidence is insufficient for identifying transmission clusters, regardless of the algorithm employed. Accordingly, neither the Poisson early-warning algorithm nor the 2-case algorithm performed significantly better than chance in detecting transmission events. Patients had often changed wards or been discharged and readmitted between probable acquisition and diagnosis.

Identification of infectious disease transmission, regardless of the microbe, relies on both identifying a plausible epidemiological link and a microbiological kinship at some level of resolution. The nature of *C. difficile* transmission, with spores that can survive for long periods and a delay between acquisition and symptomatic disease, makes it difficult to initiate the investigation at the epidemiological end. In principle, all patients admitted to a hospital, as well as previously admitted patients some time back, have a plausible epidemiological link to one another. Therefore, we suggest that routine CDI surveillance of transmission should be based primarily on microbiological typing. Because there is a tradeoff for such methods regarding turnaround time and cost versus resolution, we believe that both methods are needed: one that is fast and inexpensive with low resolution (eg, HMW typing) that can be used to quickly rule out transmission, and one that may be slower and more expensive but provides high-resolution results based on WGS to confirm transmission. Ideally HMW typing should be performed at a local laboratory and WGS at a national level to enable comparable surveillance of inter- and intrahospital transmissions by performing core-genome sequence typing. In contrast, methods with intermediate turnaround time, cost, and resolution, such as PCR ribotyping, multilocus-sequence typing, pulsed-field gel electrophoresis, and restriction endonuclease analysis add little value in the context. They are neither fast enough to quickly rule out transmission, nor discriminative enough to confirm it.

If surveillance based on ward incidence is still performed to identify possible outbreaks, it should include the total ward history of the patients and should not focus solely on the test-ordering ward. It is laborious to collect ward history data for every patient manually, but this could be automated using computer software that collects data from different information systems. For instance, an algorithm could be devised for alerts when a certain number of patients with recent care at a given ward have been diagnosed with CDI. Such a solution might perform better than the ones evaluated in this study, although that remains to be investigated.

A study in the United Kingdom performed during 2007–2011^1^ showed that ∼10% of CDI cases in a hospital could be traced to a previous symptomatic case with a close hospital contact. We found lower levels of transmitted cases, and high strain diversity, in line with a previous study in a similar setting.^7^ However, only transmission within the same ward and within 30 days were captured in our study design, and no transmission from or to asymptomatic carriers were regarded. Thus, the true number of transmission events is likely higher. Additionally, transmissions from cases before the study started were not captured, and our study period was shorter than that of the study by Eyre et al.^1^

A strength of our study is that we collected all clinically diagnosed cases during 2 years at 2 laboratories, and we were able to culture and type the isolates in most cases. The diversity of typed strains was similar to the distribution of ribotypes in yearly national collections,^4^ if the lower resolution of HMW typing compared to PCR ribotyping is taken into account. We believe that this method provides representative data for our setting, that is, a setting with a low RT027 incidence with a high diversity of strains where overt outbreaks are rare.

Our study also had several limitations. As previously mentioned, the design did not allow us to detect transmissions between wards, at other units than wards, or over longer periods. Additionally, transmission from asymptomatic carriers, which has been suggested as a possibly important reservoir,^17^ could not be captured in our study design. Lastly, these results are not transferable to a high-RT027–incidence setting because only 0.3% of cases had the HMW type corresponding to this ribotype.

The high incidence of CDI in Sweden is likely due to reasons other than frequent hospital transmission between symptomatic patients. Still, transmission happens regularly and could at any time result in larger clusters and overt outbreaks, especially if high-virulence strains are introduced. To act early in the event of an outbreak, good tools for local and regional surveillance of *C. difficile* are needed. Patients change wards, and we have shown that individual patient ward history must be considered when identifying possible transmission events. For the best possible surveillance of outbreaks, we suggest that fast methods, such as HMW typing, are used in conjunction with WGS to make it possible both to quickly rule out transmission and to reliably confirm it.
